# Assessment of Clinical Benefit of Integrative Genomic Profiling in Advanced Solid Tumors

**DOI:** 10.1001/jamaoncol.2020.7987

**Published:** 2021-02-25

**Authors:** Erin F. Cobain, Yi-Mi Wu, Pankaj Vats, Rashmi Chugh, Francis Worden, David C. Smith, Scott M. Schuetze, Mark M. Zalupski, Vaibhav Sahai, Ajjai Alva, Anne F. Schott, Megan E. V. Caram, Daniel F. Hayes, Elena M. Stoffel, Michelle F. Jacobs, Chandan Kumar-Sinha, Xuhong Cao, Rui Wang, David Lucas, Yu Ning, Erica Rabban, Janice Bell, Sandra Camelo-Piragua, Aaron M. Udager, Marcin Cieslik, Robert J. Lonigro, Lakshmi P. Kunju, Dan R. Robinson, Moshe Talpaz, Arul M. Chinnaiyan

**Affiliations:** 1 Department of Internal Medicine, University of Michigan, Ann Arbor; 2 Michigan Center for Translational Pathology, University of Michigan, Ann Arbor; 3 Department of Pathology, University of Michigan, Ann Arbor; 4 Rogel Cancer Center, University of Michigan, Ann Arbor; 5 Department of Urology, University of Michigan, Ann Arbor; 6 Howard Hughes Medical Institute, University of Michigan, Ann Arbor

## Abstract

**Importance:**

Use of next-generation sequencing (NGS) to identify clinically actionable genomic targets has been incorporated into routine clinical practice in the management of advanced solid tumors; however, the clinical utility of this testing remains uncertain.

**Objective:**

To determine which patients derived the greatest degree of clinical benefit from NGS profiling.

**Design, Setting, and Participants:**

Patients in this cohort study underwent fresh tumor biopsy and blood sample collection for genomic profiling of paired tumor and normal DNA (whole-exome or targeted-exome capture with analysis of 1700 genes) and tumor transcriptome (RNA) sequencing. Somatic and germline genomic alterations were annotated and classified according to degree of clinical actionability. Results were returned to treating oncologists. Data were collected from May 1, 2011, to February 28, 2018, and analyzed from May 1, 2011, to April 30, 2020.

**Main Outcomes and Measures:**

Patients’ subsequent therapy and treatment response were extracted from the medical record to determine clinical benefit rate from NGS-directed therapy at 6 months and exceptional responses lasting 12 months or longer.

**Results:**

During the study period, NGS was attempted on tumors from 1138 patients and was successful in 1015 (89.2%) (MET1000 cohort) (538 men [53.0%]; mean [SD] age, 57.7 [13.3] years). Potentially clinically actionable genomic alterations were discovered in 817 patients (80.5%). Of these, 132 patients (16.2%) received sequencing-directed therapy, and 49 had clinical benefit (37.1%). Exceptional responses were observed in 26 patients (19.7% of treated patients). Pathogenic germline variants (PGVs) were identified in 160 patients (15.8% of cohort), including 49 PGVs (4.8% of cohort) with therapeutic relevance. For 55 patients with carcinoma of unknown primary origin, NGS identified the primary site in 28 (50.9%), and sequencing-directed therapy in 13 patients resulted in clinical benefit in 7 instances (53.8%), including 5 exceptional responses.

**Conclusions and Relevance:**

The high rate of therapeutically relevant PGVs identified across diverse cancer types supports a recommendation for directed germline testing in all patients with advanced cancer. The high frequency of therapeutically relevant somatic and germline findings in patients with carcinoma of unknown primary origin and other rare cancers supports the use of comprehensive NGS profiling as a component of standard of care for these disease entities.

## Key Points

**Question:**

What is the clinical utility of genomic profiling for patients with advanced solid tumors?

**Findings:**

In this cohort study of 1015 patients who underwent integrative genomic profiling, a high rate of pathogenic germline variants and a subset of patients who derive substantial clinical benefit from sequencing information were identified.

**Meaning:**

These findings support (1) directed germline testing for inherited cancer predisposition in all patients with advanced cancer and (2) use of integrative genomic profiling as a component of standard of care for patients with cancer of unknown origin and other rare malignant neoplasms.

## Teaser

This cohort study assesses which patients with advanced solid tumors derive the greatest clinical benefit from next-generation sequencing.

## Introduction

Identification of tumor genomic alterations predictive of therapeutic benefit from targeted therapy has improved clinical outcomes across a wide range of malignant neoplasms.^1,2,3,4,5,6,7^ Examples include *EGFR*-mutant non–small cell lung cancer treated with epidermal growth factor receptor tyrosine kinase inhibitors, *ERBB2* (formerly *HER2/neu*)-amplified breast cancer treated with human epidermal growth factor receptor 2–specific antibodies, and tumors with microsatellite instability treated with immune checkpoint blockade. Because identification of these targets susceptible to drug therapy has improved clinical outcomes, interest in using next-generation sequencing (NGS) testing for advanced solid tumors to identify patients who qualify for enrollment in biomarker-selected clinical trials is increasing. Next-generation sequencing testing can be obtained via commercial or institutional platforms, and these differ significantly in their scope; some are limited to DNA-based testing of a select group of known, targetable cancer-related genes. Others use unbiased, comprehensive DNA- and RNA-based analyses of samples consisting of tumor and normal samples, with additional goals of identifying novel somatic alterations or pathogenic germline variants (PGVs) conferring increased cancer risk.

Studies of comprehensive NGS testing in patients with advanced cancer^8,9,10,11,12,13,14,15,16^ report a wide range of clinically actionable genomic alterations per patient, ranging from 40% to 94%. Furthermore, many studies^15,16,17^ note that only 10% to 25% of patients receive therapy informed by sequencing, making it challenging to assess the degree of clinical benefit gained. The only randomized clinical trial to explore the clinical effects of delivering genomically directed therapy to patients undergoing NGS testing in the setting of advanced cancer found no improvement in progression-free survival for patients receiving molecularly matched therapy.^18^ Other nonrandomized studies^11,15,17,19,20,21,22,23^ have demonstrated a modest clinical benefit from delivery of molecularly targeted therapy; however, interpretation of these results is often confounded by use of end points in which patients serve as their own control, such as comparison of progression-free survival during targeted therapy with progression-free survival during the patient’s previous line of therapy.

In this study, we sought to conduct a comprehensive analysis of the clinical outcomes of patients with advanced solid tumors who had NGS performed on metastatic tissue through participation in a prospective clinical cohort study, the Michigan Oncology Sequencing Program (Mi-ONCOSEQ). Robinson et al^12^ previously summarized the molecular findings from the first 500 patients enrolled, which determined that PGVs were identified at a high rate (12.2%) and that DNA and RNA sequencing were both contributory in identifying clinically actionable targets. Herein, with an expanded cohort of more than 1000 patients linked to clinical outcomes, we determine which patients derived the greatest clinical benefit from sequencing-directed therapy (SDT).

## Methods

### Study Description

Mi-ONCOSEQ was established in 2011 at the Michigan Center for Translational Pathology^24^ and is a Clinical Laboratory Improvement Amendments–certified laboratory conducting integrative sequencing of tumor and normal (blood or buccal swab) specimens. Eligibility included advanced or metastatic cancer, being 18 years or older, and the ability to safely undergo fresh tumor sampling by imaging-guided biopsy. All patients provided written informed consent, which included willingness to receive information regarding PGVs if identified. This study was approved by the institutional review board of the University of Michigan and followed the College of American Pathologists reporting guideline.

### Integrative Clinical Sequencing

Needle biopsy samples or surgically resected tissue samples were flash frozen, and a section was cut for evaluation. Remaining portions of each specimen were retained for nucleic acid extraction. Hematoxylin-eosin–stained frozen sections were reviewed by study pathologists (D.L., S.C.-P., A.M.U., and L.P.K.) to identify areas with highest tumor content. If no tumor was identified, an archival formalin-fixed paraffin-embedded sample was obtained for sequencing if available. Nucleic acid preparation and integrative clinical sequencing were performed using standard protocols.^12^ A subset of tumor samples was analyzed using a targeted panel of 1700 genes (eTable 1 in Supplement 1). Use of a whole-exome or a targeted panel for each case is described in eTable 2 in Supplement 2. Germline variants were annotated using published literature and public databases (ClinVar and the Human Genome Mutation Database), with pathogenicity determined as per American College of Medical Genetics and Genomics guidelines.^25^ Only variants previously described as pathogenic or likely pathogenic were disclosed. Sequencing data from all patients can be obtained from the Database of Genotypes and Phenotypes under accession number phs000673.v4.p1.

### Carcinoma of Unknown Primary Tissue of Origin Estimation

Tissue from a carcinoma of unknown primary (CUP) origin estimation was implemented using a published method,^26^ but adapted for RNA-sequencing data with modifications. For training, the Cancer Genome Atlas data of primary tumor tissues were artificially contaminated with expression from normal tissues (obtained from the Genotype-Tissue Expression program, the Cancer Genome Atlas, and the Human Protein Atlas). Each estimation was based on an ensemble learning approach aggregating 6 different models (bootstrap aggregation; each model has the same weight). The 6 models were derived from the combination of nu-support vector machine and multinomial lasso classifier machine learning algorithms and 3 ways of contaminating training examples (none, expression from the normal biopsy site, and mean model of expression from all possible biopsy sites).

### Precision Medicine Tumor Board and Results Disclosure

A monthly multidisciplinary precision medicine tumor board reviewed, interpreted, and discussed sequencing results for patients whose NGS results were of clinical importance (eFigure 1 in Supplement 3 and eTable 2 in Supplement 2). For all patients, summarized results were presented in the form of a report (generated within 4 to 6 weeks of patient enrollment) to treating oncologists, with opportunity to review by the precision medicine tumor board on request (eFigure 1 in Supplement 3). Considerations for targeted therapy included on reports were informed by use of variant annotation databases such as OncoKB, CIViC, My Cancer Genome, and literature review coupled with the National Comprehensive Cancer Network, US Food and Drug Administration guidelines and approvals, and ClinicalTrials.gov. For germline variants, ClinVar, as well as gene-specific databases, was consulted.

### Generation of Genomic Alteration Tiers

Genomic alterations were classified in a tumor-specific manner and placed in 1 of 3 tiers according to levels of evidence and potential for clinical action. Tier 1 and 2 genomic alterations were considered potentially clinically actionable. The Mi-ONCOSEQ genomic alteration tiers are similar to previously published schemas,^27,28,29^ with a few additions: (1) PGVs are categorized separately from somatic variants, distinguishing those that confer increased cancer risk as well as those with direct therapeutic implications; and (2) change in cancer diagnosis as determined by transcriptomic profiling is incorporated.

### Analysis of Clinical Outcomes

Data were analyzed from May 1, 2011, to April 30, 2020. Medical record reviews were performed by a medical oncologist (E.F.C.). It was determined whether a patient received SDT via review of clinician notes, which often directly referenced the Mi-ONCOSEQ report or precision medicine tumor board discussion. Sequencing-directed therapy could be delivered in clinical trials, off-label, or on-label at the discretion of the treating oncologist. Time receiving treatment and best response to therapy (progressive disease, stable disease, partial response, or complete response) were recorded. For patients treated with SDT during a clinical trial, response evaluation criteria in solid tumors (RECIST) were directly abstracted from the medical record. For patients not treated during a clinical trial, best response to therapy was determined by a medical oncologist (E.F.C.) via review of clinician notes and/or direct measurement of sites of measurable disease on computed tomographic imaging and use of RECIST criteria. Clinical benefit rate was defined as the proportion of patients receiving SDT for 6 months or longer. Exceptional responders received SDT for a duration of 12 months or longer.

## Results

### Cohort Demographics

**Figure 1.**
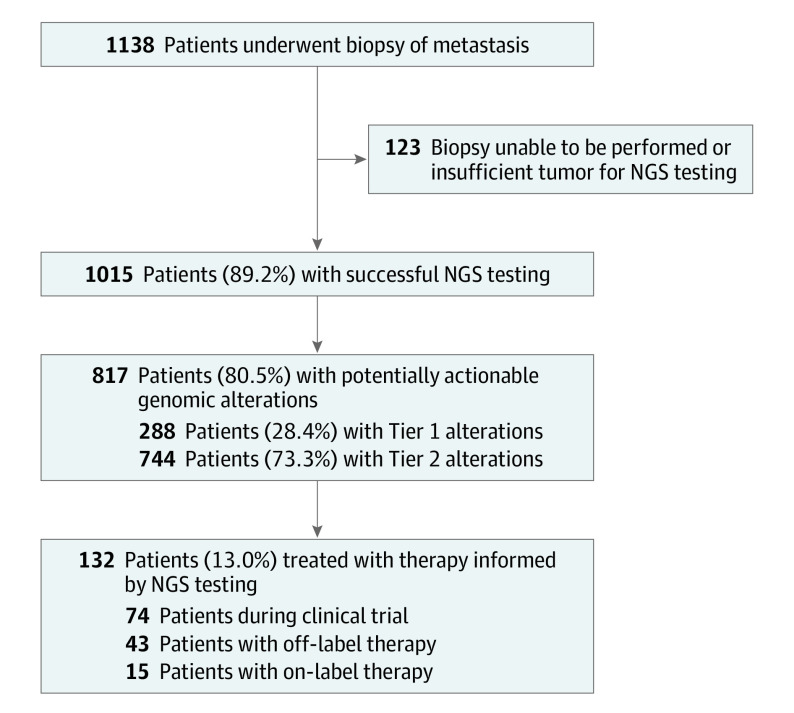
CONSORT Diagram of Patients in the MET1000 Cohort NGS indicates next-generation sequencing.

From May 1, 2011, to February 28, 2018, 1138 patients underwent biopsy for integrative clinical sequencing. Of these, 1015 had successful NGS testing (MET1000 cohort), whereas 123 did not, owing to the following: (1) patient changed consent, moved, or entered hospice before scheduled biopsy; (2) inability to safely perform planned biopsy owing to tumor location; and (3) inadequate tumor content from biopsy (Figure 1). Mean (SD) patient age at enrollment was 57.7 (13.3) years. A total of 538 patients (53.0%) were men and 477 (47.0%) were women. Types of cancers sequenced and sites of metastatic biopsy are depicted in eFigure 2 in Supplement 3. Eight hundred five patients (79.3%) received systemic therapy before enrollment (eTable 2 in Supplement 2). A mean (SD) of 47 (63) months elapsed from time of the patient’s cancer diagnosis to enrollment in Mi-ONCOSEQ (eTable 2 in Supplement 2).

### Germline and Somatic Molecular Alterations in Patients With Metastatic Solid Tumors

**Figure 2.**
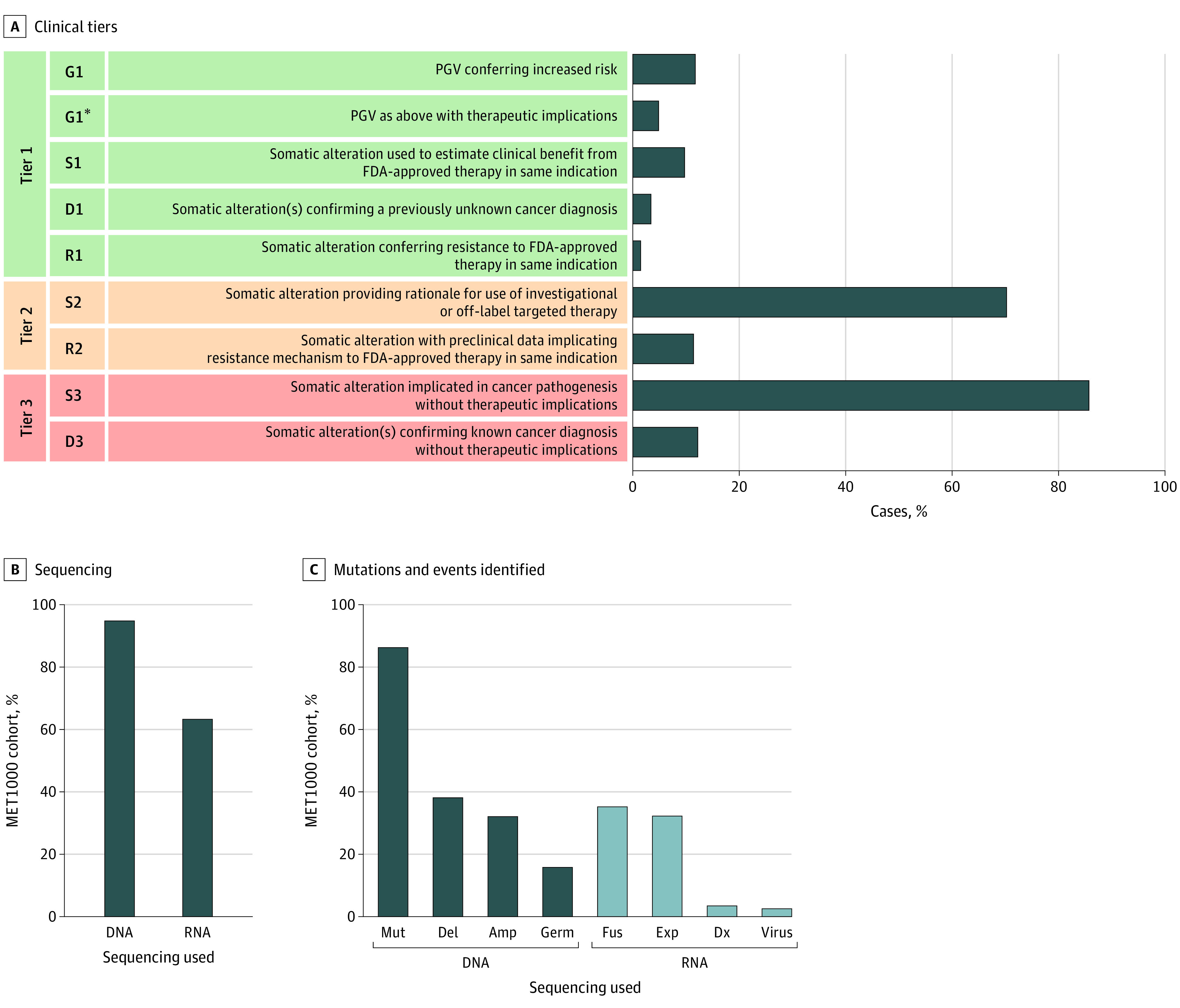
Clinical Tiering of Molecular Alterations Identified in Metastatic Cancer A, Tiering of genomic alterations identified in the MET1000 cohort by clinical relevance. D indicates diagnosis change; G, germline; R, resistance to therapy; and S, somatic. Tier 1 alterations were known to have clinical utility for that individual’s cancer type and included pathogenic germline variants (PGVs) conferring increased cancer risk, changes in cancer diagnosis, and somatic alteration(s) used to estimate clinical benefit from or resistance to a therapy approved by the US Food and Drug Administration (FDA). Tier 2 alterations included somatic events that provided rationale for use of investigational or off-label targeted therapy and alterations postulated from strong preclinical evidence to estimate resistance to an FDA-approved therapy in that indication. Tier 3 included alterations implicated in cancer pathogenesis or molecular events indicative of a known cancer diagnosis but without current therapeutic implications. Genomic alterations in tiers 1 and 2 were considered potentially clinically actionable. B, Percentage of cases in which DNA or RNA sequencing contributed to identifying clinically relevant alterations. C, Classes of clinically relevant alterations identified in the MET1000 cohort. Amp indicates amplification; Del, homozygous deletion; Dx, markers for cancer of unknown primary origin or change of diagnosis; Exp, expression concordant with gene amplification; Fus, gene fusion; Germ, germline; Mut, mutation; and Virus, viral pathogen.

Among 1015 tumors sequenced, 817 (80.5% of cohort) harbored at least 1 potentially actionable alteration, classified as tier 1 or 2 (Figure 2A). Tumors in 713 patients (70.2%) harbored a somatic molecular alteration that was potentially actionable and could provide rationale for the use of investigational targeted therapy or off-label therapy (designated tier 2 S2). Among tier 1 and 2 molecular alterations, 962 (94.8% of total) were identified by DNA sequencing and 645 (63.5% of total) were identified by RNA sequencing (Figure 2B). Integrative sequencing revealed a variety of alteration classes; DNA sequencing of tumor and normal samples identified mutations, amplifications, homozygous deletions, and germline alterations (Figure 2C). RNA sequencing complemented this analysis and was essential in the identification of 103 clinically actionable molecular events (10.1% of total), particularly with regard to identification of oncogenic gene fusions (Figure 2C). RNA sequencing also provided information on outlier expression concordant with amplification, viral pathogens, and markers for CUP or change of diagnosis. In terms of the contribution of each mode of sequence analyses (mutation, copy number determination, and RNA-seq analysis) to the yield of informative aberrations, in 579 cases (57.0%), informative alterations were detected by all of the 3 modes of integrated sequencing and analysis. In 213 cases (21.0%), clinically relevant aberrations were detected by mutation and copy number analyses, but no reportable aberrations were detected by RNA-seq. In 51 cases (5.0%), only RNA-seq yielded clinically informative events, emphasizing the increased clinical yield of the integrated sequence and analyses approach used here. Clinical tiering information for all molecular events identified in the MET1000 cohort is detailed in eTable 3 in Supplement 4.

### Use of SDT

**Figure 3.**
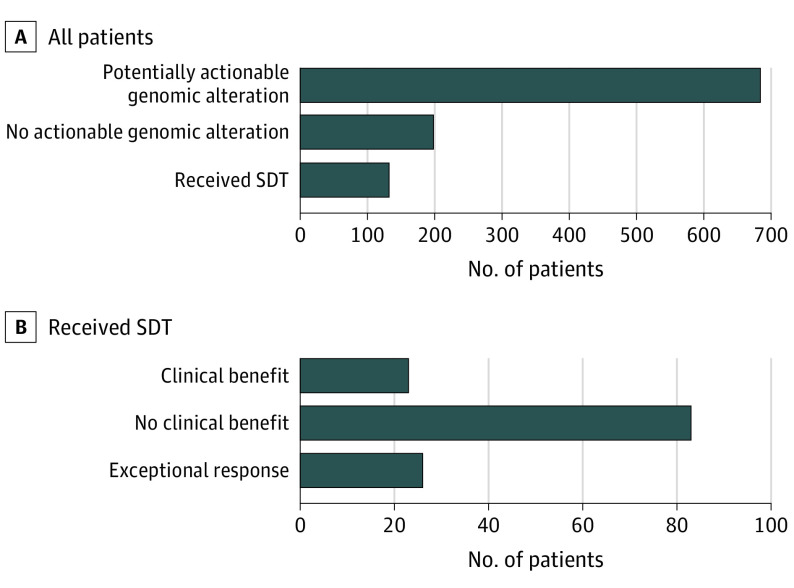
Patients Receiving Sequencing-Directed Therapy (SDT) in MET1000 Cohort and Exceptional Responses Bar graphs depict proportion of patients in the MET1000 cohort (n = 1015) who received SDT and ultimately had clinical benefit or exceptional response to treatment.

Sequencing-directed therapy was initiated in 132 patients (16.2% of 817 patients with clinically actionable alterations) (Figure 3). Median time from study enrollment to initiation of SDT was 3.8 (range, 0.2-44.0) months. Among those receiving SDT, 74 patients were treated during a clinical trial, 43 with off-label therapy, and 15 with on-label therapy (Figure 1; eTable 4 in Supplement 5). Of these, 49 patients (37.1% of those receiving SDT) experienced clinical benefit. The most common cancer types with clinical benefit from SDT were sarcoma (12 of 138 [8.7%]), prostate adenocarcinoma (10 of 154 [6.5%]), and CUP (7 of 55 [12.7%]). Most patients receiving SDT (93 [70.5%]) were treated with targeted small-molecule inhibitors, including CDK4/6 inhibitors in 21 patients, inhibitors of poly–adenosine diphosphate ribose polymerase (PARPis) in 16 patients, and inhibitors of fibroblast growth factor receptor in 11 patients. Additional common SDTs included an immune checkpoint inhibitor (ICI) in 29 patients (22.0%), monoclonal antibodies against human epidermal growth factor receptor 2 in 8 patients (6.1%), and antiestrogen therapy in 2 patients (1.5%).

### Patients With Exceptional Response to SDT

Twenty-six patients (19.7% of those receiving SDT) received treatment for 12 months or longer (Figure 3, eTable 4 in Supplement 5, and eFigures 3-4 in Supplement 3), ranging from 12.1 to 39.5 months. Genomic alterations in those with an exceptional response included 10 cases with defects in DNA repair, of whom 5 had double-strand DNA repair defects (*BRCA1*/*BRCA2* [OMIM 113705/OMIM 600185]: 4 somatic and 1 PGV) and 5 had defects in DNA mismatch repair (*MSH2* [OMIM 609309]: 3 somatic and 2 PGVs). Five cases harbored driver gene fusions, including a novel fusion involving the progesterone receptor (*PGR*) in a patient who developed chondrosarcoma during pregnancy and had an exceptional response to tamoxifen. Three patients with exceptional responses had tumors with activating hotspot mutations, treated with targeted small-molecule inhibitors. Five cases with amplifications in the CDK4/6 pathway had exceptional response when treated with CDK4/6 inhibitors, and 1 patient with CUP had an exceptional response to dual human epidermal growth factor receptor 2 monoclonal antibody treatment, which was initiated based on *ERBB2* amplification. In 2 cases, 1 with breast cancer and 1 with CUP, patients had exceptional responses to programmed cell death protein inhibitor therapy, which was initiated based on high mutation burden by a mechanism other than microsatellite instability (eFigure 3 in Supplement 3). The most common reason for discontinuation of SDT was disease progression (eTable 4 in Supplement 5). Three patients receiving ICI therapy based on high mutation burden by mechanism other than microsatellite instability or alternative mechanism of high mutation burden achieved a complete response and remain without evidence of disease.

### Pathogenic Germline Variants

**Figure 4.**
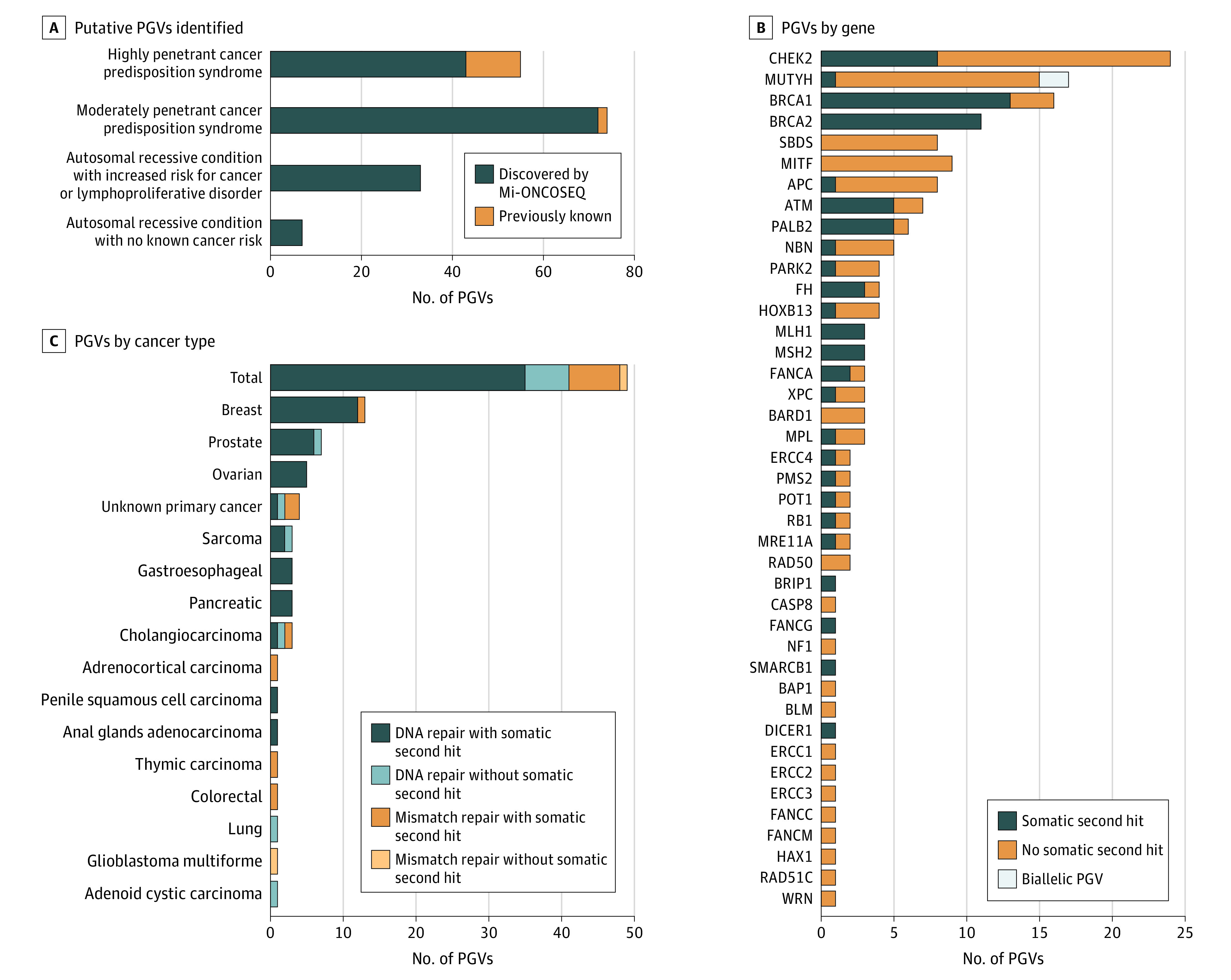
Pathogenic Germline Variants (PGVs) Observed in the MET1000 Cohort A, Among 1015 patients undergoing sequencing, 169 putative PGVs were identified (160 patients [15.8%] of MET1000 cohort). Fifty-five PGVs associated with highly penetrant cancer predisposition syndromes (including PGVs in *APC, BAP1, BRCA1, BRCA2, DICER1, FH, HOXB13, MLH1, MSH2, PALB2, PMS2, POT1,* and *RB1*) were identified, which included 14 PGVs known before the patient’s enrollment in the Michigan Oncology Sequencing Program (Mi-ONCOSEQ). Seventy-four PGVs associated with moderately penetrant cancer predisposition syndromes (including PGVs in *APC, ATM, BARD1, CHEK2, MITF, MRE11A, MUTYH, RAD50, RAD51C, NF1,* and *SMARCB1*) were identified, which included 2 PGVs known before the patient’s enrollment in Mi-ONCOSEQ. An additional 33 PGVs associated with an autosomal recessive condition conferring increased risk for cancer or lymphoproliferative disorder (including *MPL, BLM, CASP8, ERCC1, ERCC2, ERCC3, ERCC4, FANCA, FANCC, FANCG, FANCM, HAX1, NBN, SBDS,* and *XPC*) and 7 PGVs associated with an autosomal recessive condition not known to increase cancer risk (including *PARK2, FH,* and *WRN*) were identified. B, Pathogenic germline variants with somatic second-hit events identified by gene. Sixty-nine of the PGVs identified (40.8% of PGVs and 6.8% of MET1000 cohort) harbored a somatic second-hit event in the tumor. Incidental PGVs in highly penetrant cancer predisposition syndromes (ie, *BRCA1*) were identified in cases where the PGV was not likely related to tumor pathogenesis. C, Pathogenic germline variants with therapeutic targets were identified in 49 patients (4.8% of MET1000 cohort), often in diseases not typically associated with cancer predisposition syndromes in DNA or mismatch repair. Among the 49 PGVs identified with a therapeutic target, 42 (85.7%) harbored a somatic second-hit event in the tumor.

A total of 169 PGVs were identified among 160 unique patients (15.8% of cohort), 49 of whom (4.8% of cohort) were designated as having potential therapeutic implications (tier 1, G1*) (Figure 2A and eTable 5 in Supplement 6). Most PGVs (155 of 169 [91.7%]) were unknown before enrollment in Mi-ONCOSEQ and were classified as belonging to 1 of 4 categories shown in Figure 4A (eTable 5 in Supplement 6). One hundred fifteen PGVs were identified (68.0% of total PGVs and 11.3% of cohort) that were indicative of highly or moderately penetrant cancer predisposition syndromes. Sixty-nine of 169 PGVs (40.8%) harbored a somatic second hit event in the tumor (Figure 4B). Pathogenic germline variants associated with therapeutic targets, such as those resulting in defects in double-strand DNA repair (*BRCA1, BRCA2, ATM, PALB2,* and *BRIP1)* or DNA mismatch repair (*MLH1, MSH2,* and *PMS2*), were identified in 49 patients (29.0% of total PGVs and 4.8% of cohort), 37 of which had not been identified before enrollment. Fourteen PGVs in DNA double-strand repair and 7 PGVs in DNA mismatch repair were identified in cancer types not commonly associated with hereditary breast and ovarian cancer or Lynch syndromes, including CUP and sarcomas (Figure 4C). Seven patients received a PARPi, 3 received an ICI, and 1 received both PARPi and ICI therapy owing to identification of PGVs in DNA repair. Six of these patients achieved clinical benefit from treatment (eFigure 5 in Supplement 3).

### CUP Origin

**Figure 5.**
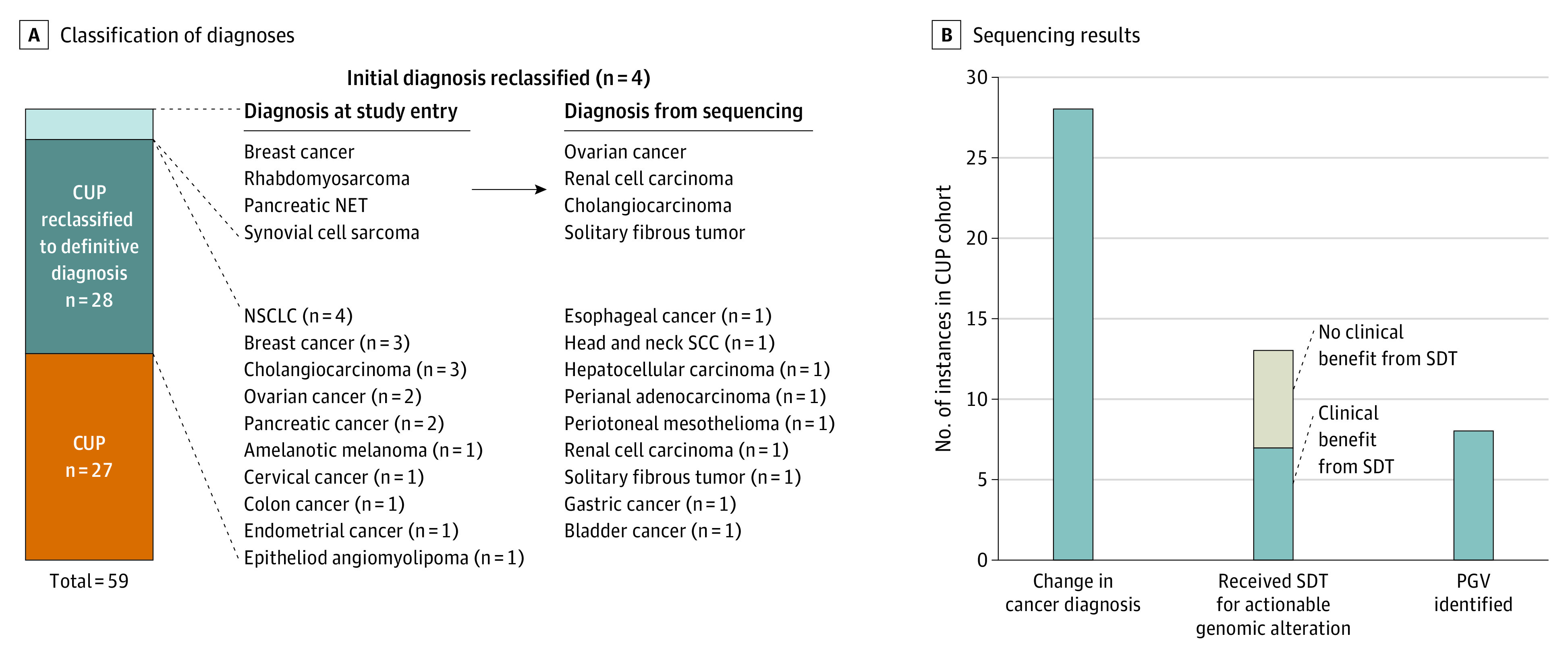
Cancers of Unknown Primary Origin in MET1000 Cohort A, Among 55 cases of cancer of unknown primary (CUP) origin sequenced, 28 (50.9%) were reclassified to a definitive diagnosis through RNA sequencing tissue of origin predictor. An additional 4 cases in the MET1000 cohort with presumed known diagnoses at study entry were also reclassified. B, Sequencing results were highly informative for patients with CUP, with a total of 34 of 55 CUP cases (61.8%) having at least 1 of the following: (1) a change in cancer diagnosis (28 patients [50.9%]), (2) receipt of sequencing-directed therapy (SDT) (13 patients [23.6%]), or (3) identification of a pathogenic germline variant (PGV) conferring increased cancer risk (8 patients [14.5%]). NET indicates neuroendocrine tumor; NSCLC, non–small cell lung cancer; and SCC, squamous cell carcinoma.

Before NGS testing, 55 patients had a diagnosis of CUP. The median number of prior lines of systemic therapy for patients with CUP at Mi-ONCOSEQ enrollment was 0, ranging from 0 to 4 (eTable 6 in Supplement 3). Twenty-eight CUP cases (50.9%) were reclassified to an alternative cancer diagnosis, whereas a definitive diagnosis was not able to be established for the remaining 27 cases. In addition, 4 more cancers that were initially classified as originating from a specific tissue were reclassified using this same method (Figure 5A and eTable 6 in Supplement 3). Sequencing-directed therapy was initiated in 13 instances for patients who initially had CUP (23.6%); 6 patients experienced a clinical benefit, including 1 who had clinical benefit from 2 SDTs administered serially (clinical benefit rate, 53.8%). Five exceptional responses were observed (Figure 5B and eFigure 3 in Supplement 3). Pathogenic germline variants were identified in 8 patients with CUP (14.5%), 4 of which were therapeutically relevant (*BRCA1* in 2 patients, *BRCA2* in 1 patient, and *MSH2* in 1 patient) (eTable 5 in Supplement 6).

## Discussion

In this study, we found that combined genomic profiling of tumor and normal specimens was a clinically powerful tool. Most importantly, a high incidence of PGVs was observed, approximately 1 in every 6 patients, with many of the PGVs identified having therapeutic relevance and resulting in clinical benefit when targeted therapies were administered. In addition, this finding has significant implications for patients’ family members, who can be offered genetic testing and enhanced screening or risk-reducing interventions if a PGV is identified. Our study also found that patients with CUP derived significant clinical benefit from NGS profiling. At present, CUP has no clear standard treatment paradigm. Among this cohort, 50.9% of CUP tumors were reclassified to a specific cancer diagnosis, allowing some patients to receive standard of care therapy for that disease entity and 13 (23.6%) to receive SDT. Furthermore, a high proportion of these patients harbored PGVs (14.5%).

Among all patients treated with SDT, 37.1% had clinical benefit from treatment, including 19.7% with exceptional response. None of these therapies would have been recommended per standard of care guidelines, indicating that sequencing information was of significant value. The most common genomic alterations portending exceptional response included defects in DNA double-strand repair and DNA mismatch repair, for which patients received either PARPi or ICI. In many circumstances, DNA repair defects were identified in tumor types not commonly associated with these genomic changes, such as CUP and cholangiocarcinoma. Last, some patients achieved exceptional response to targeted therapy administered on the basis of a driver gene fusion, which would not have been identified without RNA sequencing.

Assessing the clinical utility of NGS testing in oncology remains challenging for several reasons: (1) the definition of clinically actionable alterations is dynamic as new therapeutics emerge; (2) patients’ tumors often harbor numerous alterations, with uncertainty surrounding which events are most important to target; (3) patients with advanced malignant neoplasms and multiple prior therapies may not qualify for trial enrollment or may tolerate treatment poorly; (4) clinical behavior of diverse cancer types included in studies is highly variable; and (5) availability of biomarker-selected trials may be limited, and insurance may not cover off-label therapies. Indeed, these challenges highlight the need to explore novel clinical trial end points and develop large-scale precision oncology studies with access to a wide range of targeted therapies. Two of the largest clinical trials using this strategy, Molecular Analysis for Therapy Choice (MATCH)^30,31,32^ and Targeted Agent and Profiling Utilization Registry (TAPUR),^33,34^ are ongoing. However, few studies are specifically exploring the effect of identifying incidental PGVs from tumor NGS testing.

### Strengths and Limitations

Strengths of this study include the use of DNA and RNA sequencing in addition to assessment of tumor and normal specimens, allowing for identification of previously unidentified PGVs, gene fusions, and tissue of origin estimation. Additional strengths are inclusion of large numbers of patients with rare cancers and clinical outcomes analyses for patients treated with SDT. Limitations are that clinical outcome analyses are retrospective and largely descriptive in nature with lack of a comparator or control population to quantify the degree of clinical benefit achieved by testing. However, the diversity of patients, tumor types, and molecular characteristics included within this study make identification of an appropriate control population difficult. Furthermore, the proportion of patients who received SDT is low (13.0%) and in accordance with rates previously reported in studies, which have used more limited sequencing approaches (ie, targeted DNA-based panels),^16,21,35^ indicating that broader sequencing approaches have not yet translated into improved ability to match patients with targeted therapy. Despite this, we observed that our ability to direct patients toward clinical trials of targeted therapy improved over time (eTable 7 in Supplement 3). Last, our study does not explore other sequencing methods that may be of clinical or scientific value, such as analysis of liquid biopsy specimens in the form of circulating tumor DNA or novel tumor-based sequencing approaches such as methylome profiling.

## Conclusions

Our data support a recommendation for germline testing of DNA repair genes as standard practice in patients with metastatic solid tumors and comprehensive NGS profiling at diagnosis for patients with CUP. With continued discovery of genomic biomarkers predictive of clinical benefit from anticancer therapies, we anticipate even broader clinical applicability of this technology.

## Notes

Supplement 1. **eTable 1.** Gene list of the Mi-ONCOSEQ Targeted Panel

Supplement 2. **eTable 2.** MET1000 Cohort Demographics and Sequencing Information

Supplement 3. **eFigure 1.** Sample Mi-ONCOSEQ Patient Report **eFigure 2.** Diverse Metastatic Cancer Types Represented in the MET1000 Cohort and Biopsy Sites **eFigure 3.** Time on Treatment for Patients With Exceptional Response to Sequencing-Directed Therapy **eFigure 4.** Baseline and Subsequent Computed Tomography Images From 4 Patients Who Had Exceptional Response to Sequencing-Directed Therapy **eFigure 5.** Time on Treatment for Patients Receiving Therapy Based on a Pathogenic Germline Variant **eTable 6.** Detailed Information Regarding Patients With CUP in the MET1000 Cohort **eTable 7.** Proportion of Patients Receiving Sequencing-Directed Therapy by Year of Enrollment

Supplement 4. **eTable 3.** Informative Molecular Events Identified in the MET1000 Cohort

Supplement 5. **eTable 4.** Detailed Treatment Information for Patients in MET1000 Cohort Receiving Sequencing-Directed Therapy

Supplement 6. **eTable 5.** Detailed Information Regarding Pathogenic Germline Variants Identified in the MET1000 Cohort
