# Supplementary material for: Assessment of Clinical Benefit of Integrative Genomic Profiling in Advanced Solid Tumors
Source: JAMA Oncol. 2021 Feb 25;7(4):1–9. doi: 10.1001/jamaoncol.2020.7987 (PMC7907987; doi:10.1001/jamaoncol.2020.7987)
Supplement: Supplement 3. — eFigure 1. Sample Mi-ONCOSEQ Patient Report eFigure 2. Diverse Metastatic Cancer Types Represented in the MET1000 Cohort and Biopsy Sites eFigure 3. Time on Treatment for Patients With Exceptional Response to Sequencing-Directed Therapy eFigure 4. Baseline and Subsequent Computed Tomography Images From 4 Patients Who Had Exceptional Response to Sequencing-Directed Therapy eFigure 5. Time on Treatment for Patients Receiving Therapy Based on a Pathogenic Germline Variant eTable 6. Detailed Information Regarding Patients With CUP in the MET1000 Cohort eTable 7. Proportion of Patients Receiving Sequencing-Directed Therapy by Year of Enrollment [file jamaoncol-e207987-s003.pdf]

Supplementary Online Content

Cobain EF, Wu Y-M, Vats P, et al. Assessment of clinical benefit of integrative genomic profiling in advanced solid tumors. *JAMA Oncol.* Published online February 25, 2021. doi:10.1001/jamaoncol.2020.7987

eFigure 1. Sample Mi-ONCOSEQ Patient Report
eFigure 2. Diverse Metastatic Cancer Types Represented in the MET1000 Cohort and Biopsy Sites
eFigure 3. Time on Treatment for Patients With Exceptional Response to Sequencing-Directed Therapy
eFigure 4. Baseline and Subsequent Computed Tomography Images From 4 Patients Who Had Exceptional Response to Sequencing-Directed Therapy
eFigure 5. Time on Treatment for Patients Receiving Therapy Based on a Pathogenic Germline Variant
eTable 6. Detailed Information Regarding Patients With CUP in the MET1000 Cohort
eTable 7. Proportion of Patients Receiving Sequencing-Directed Therapy by Year of Enrollment

This supplementary material has been provided by the authors to give readers additional information about their work.

©2021 Cobain EF. *JAMA Oncology*.

eFigure 1. Sample Mi-ONCOSEQ Patient Report

MICHIGAN MEDICINE
UNIVERSITY OF MICHIGAN
MICHIGAN CENTER FOR TRANSLATIONAL PATHOLOGY

Patient ID: TP_2257

ONCOSEQ INTEGRATIVE SEQUENCING EXECUTIVE REPORT

PATIENT INFORMATION

**Name:**		**Age/Gender:**	53 y/o male	**DOB:**	08-27-1962
**Ethnic Background:**	Caucasian	**Medical Record #:**		**PATIENT ID:**	TP_2257
**Submitted Diagnosis:**	Metastatic sarcomatoid carcinoma of unknown primary	**Submitting Pathologist:**			
**Treating Physician:**		**Submitted Specimen Site (tumor):**	Colon and omentum	**Submitted Specimen Site (normal):**	Blood
**PMTB Date:**	Not in PMTB	**Specimen Type (tumor):**	FFPE, Block A1	**Specimen Type (normal):**	Ambient
**Report Date:**	September 2016	**Collection Method (tumor):**	Resection	**Collection Method (normal):**	Blood draw
**Molecular Analysis by:**		**Specimen Collection Date (tumor):**	June 2016	**Specimen Collection Date (normal):**	August 2016
**Relevant history:**	CURRENT TREATMENT: None<br>PAST TREATMENT: None<br>6/2016: Doing well until the beginning of June when he became fatigued. He was still able to go to work and do chores but noticed he'd become wiped out after activities such as mowing the lawn. He described often "not feeling quite right" but had no specific symptom.<br>6/14/2016: Developed acute onset severe abdominal pain. CT scan showed a large mass in the left upper quadrant.<br>6/16/2016: Exploratory surgery found a tumor the size of volleyball in his left upper quadrant as well as a large amount of ascites. The tumor was resected in whole; however, after removal of the primary, there was a residual spot of tumor on his abdominal wall. His pathology report reviewed at U of M was metastatic sarcomatoid carcinoma of unknown primary.<br>7/2016: Tumor FFPE was obtained for MI-Oncoseq Sequencing Study.				

SEQUENCING QC REPORT

Libraries: Tumor and normal OncoSeq exome capture libraries and tumor whole transcriptome capture libraries were analyzed.

Sample Quality	Sequencing Quality	Library Quality	Sample Identity (SNP Fingerprinting)
**Pass**	**Pass**	**Pass**	**Pass**

POTENTIALLY ACTIONABLE/INFORMATIVE RESULTS

Mutation class	Gene/Aberration	Potential Therapies/*Clinical Trials (*Contingent on meeting study eligibility criteria)
Somatic Point Mutations (Total: 57)<br>**20.1 Mutations/Mb**	**TP53: p.R342*, LOH**<br>**SMARCA4: p.T910M, LOH** (compromised ATPase activity)<br>55 additional point mutations of uncertain significance are listed in the Supplementary Table	High mutation burden: immune checkpoint inhibitors<br>NCT02465060, NCT01876511, NCT02628067, NCT02646748
Somatic Indels (Total: 8)	CDK13 (p.A162fs), GLIS2 (p.G397fs), HDAC2 (p.T459fs), KMT2B (p.G1099fs), PDS5B (p.K1318fs), XIRP2 (p.S470fs), ZFHX3 (p.P3419fs), ZNF384 (p.Q453dup)	
Copy Number Aberrations	**Aneuploid**<br>**Genome-wide uniparental disomy (UPD)**<br>(See Copy number plot below)	
Gene Fusions	No driver fusion detected	
Outlier Gene Expression	No notable outliers<br>**Tumor origin remains unknown**	
Germline Variants for Disclosure	**MSH2: p.Q409fs**<br>Loss of heterozygosity (LOH) through UPD in tumor	Genetic counseling<br>Immunotherapy

INTERPRETATION OF RESULTS

- ***Aberrations that may relate to standard of care:*** N/A
- ***Aberrations that may make patient eligible for an open clinical trial or other therapies:***

OncoSeq was developed and its performance characteristics determined by the Michigan Center for Translational Pathology. It has not been cleared or approved by the FDA. The laboratory is regulated under CLIA as qualified to perform high-complexity testing. This test is used for clinical purposes.

Note: Clinical sequencing is carried out for patients enrolled on IRB approved protocols (HUM00046018; HUM00067928; HUM00056496).

CLIA#: 23D0366712 **Electronic Signature by:**

Address: 1500 E. Medical Center Drive, Ann Arbor, MI 48109; Email: MiOncoSeq-Admin@med.umich.edu; Phone: 734-763-2826; Fax: (734) 647–7827

©2021 Cobain EF. *JAMA Oncology*.

Patient ID: TP_2257

MSH2 p.Q409fs germline variant with LOH in tumor:

NCT02465060: Molecular Analysis for Therapy Choice (MATCH). Subprotocol Z1D (Loss of MLH1 or MSH2 by IHC) - Patients with mismatch repair deficiency (loss of MLH1 or MSH2 by IHC) receive nivolumab.
NCT01876511: Phase 2 study of MK-3475 in patients with Microsatellite Unstable (MSI) Tumors. MK-3475 is a humanized monoclonal antibody against human cell surface receptor PD-1.
NCT02628067: A Clinical Trial of Pembrolizumab (MK-3475) Evaluating Predictive Biomarkers in Subjects with Advanced Solid Tumors (KEYNOTE 158).
NCT02646748: A Platform Study Exploring the Safety, Tolerability, Effect on the Tumor Microenvironment, and Efficacy of Pembrolizumab + INCB Combinations in Advanced Solid Tumors. Condition: MMR-deficient Tumors.

- ***Germline variants/family history- implications for disclosure:***

MSH2 p.Q409fs variant, pathogenic

Analysis of patient's peripheral blood sample identified a pathogenic germline variant in the *MSH2* gene (NM_000251.2: c.1226_1227delAG, p.Gln409fs; dbSNP: rs63750086). Mutations of MSH2 have been previously reported in association with Lynch syndrome (also known as Hereditary Nonpolyposis Colorectal Cancer) (Ref 1-5). Lynch syndrome is an autosomal dominant cancer predisposition syndrome characterized by an increased risk of colorectal cancer and cancers of the endometrium, ovary, stomach, small intestine, hepatobiliary tract, urinary tract, brain, and skin. Screening and surveillance protocols exist which have been proven to decrease morbidity and mortality in Lynch syndrome. Genetic counseling is strongly recommended.

- ***Other informative results:***

- This tumor is deficient of mismatch repair due to combined germline and somatic inactivation of the *MSH2* gene. The hypermutation phenotype (20.1 Mutations/Mb) and observed mutational signature are consistent with defective DNA mismatch repair.

- ***References:***

1. Moslein G, Tester DJ, Lindor NM, Honchel R, Cunningham JM, French AJ, Halling KC, Schwab M, Goretzki P, Thibodeau SN. Microsatellite instability and mutation analysis of hMSH2 and hMLH1 in patients with sporadic, familial and hereditary colorectal cancer. Hum Mol Genet. 1996, 5(9):1245-52. PMID: 8872463.
2. Mangold E, Pagenstecher C, Friedl W, Mathiak M, Buettner R, Engel C, Loeffler M, Holinski-Feder E, Müller-Koch Y, Keller G, Schackert HK, Krüger S, Goecke T, Moeslein G, Kloor M, Gebert J, Kunstmann E, Schulmann K, Rüschoff J, Propping P. Spectrum and frequencies of mutations in MSH2 and MLH1 identified in 1,721 German families suspected of hereditary nonpolyposis colorectal cancer. Int J Cancer. 2005, 116(5):692-702. PMID: 15849733.
3. Tang R, Hsiung C, Wang JY, Lai CH, Chien HT, Chiu LL, Liu CT, Chen HH, Wang HM, Chen SX, Hsieh LL; TCOG HNPCC Consortium. Germ line MLH1 and MSH2 mutations in Taiwanese Lynch syndrome families: characterization of a founder genomic mutation in the MLH1 gene. Clin Genet. 2009, 75(4):334-45. PMID: 19419416.
4. Pérez-Cabornero L, Borrás Flores E, Infante Sanz M, Velasco Sampedro E, Acedo Becares A, Lastra Aras E, Cuevas González J, Pineda Riu M, Ramón y Cajal Asensio T, Capellá Munar G, Miner Pino C, Durán Domínguez M. Characterization of new founder Alu-mediated rearrangements in MSH2 gene associated with a Lynch syndrome phenotype. Cancer Prev Res (Phila). 2011, 4(10):1546-55. PMID: 21778331.
5. Sehgal R, Sheahan K, O'Connell PR, Hanly AM, Martin ST, Winter DC. Lynch syndrome: an updated review. Genes (Basel). 2014, 5(3):497-507. PMID: 24978665.

- ***Copy number and gene expression plots:***

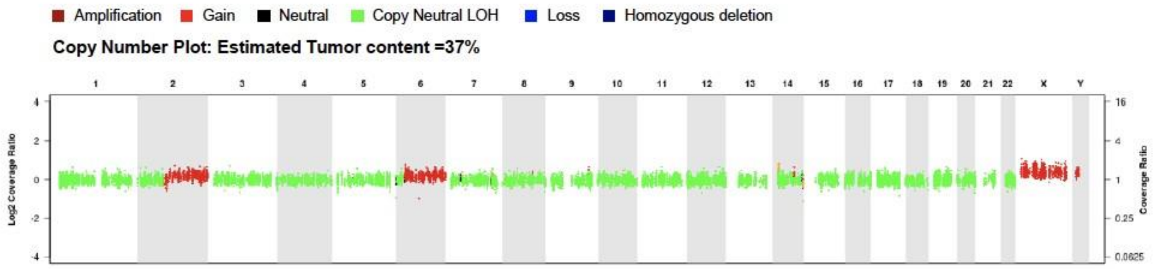

OncoSeq was developed and its performance characteristics determined by the Michigan Center for Translational Pathology. It has not been cleared or approved by the FDA. The laboratory is regulated under CLIA as qualified to perform high-complexity testing. This test is used for clinical purposes.

Address: 1500 E. Medical Center Drive, Ann Arbor, MI 48109; Email: MiOncoSeq-Admin@med.umich.edu; Phone: 734-763-2826; Fax: (734) 647–7827

©2021 Cobain EF. *JAMA Oncology*.

Patient ID: TP_2257

- ***Supplementary Tables:***

Summary of somatic mutations

Gene	Location (GRCh37)	Variant	Allelic Fraction
AATK	chr17:79095670	NM_004920.2: c.1757G>A, p.Gly586Asp	39%
ADGRL3	chr4:62812686	NM_015236.5: c.2270C>T, p.Thr757Met	32%
ARID1B	chr6:157528436	NM_017519.2: c.6122A>G, p.His2041Arg	45%
ATAD2	chr8:124338173	NM_014109.3: c.3962A>G, p.Gln1321Arg	21%
CD79B	chr17:62007182	NM_000626.3: c.497C>T, p.Thr166Met	36%
CDK13	chr7:39990716	NM_003718.4: c.484delG, p.Ala162fs	21%
EGR1	chr5:137803117	NM_001964.2: c.979C>T, p.Arg327Trp	40%
ETV7	chr6:36343801	NM_016135.3: c.154G>A, p.Ala52Thr	44%
EXT2	chr11:44228435	NM_000401.3: c.1687G>A, p.Glu563Lys	5%
FAT2	chr5:150923969	NM_001447.2: c.6719T>C, p.Val2240Ala	6%
FAT4	chr4:126408584	NM_024582.4: c.12901G>A, p.Asp4301Asn	19%
FAT4	chr4:126411664	NM_024582.4: c.13687C>G, p.Pro4563Ala	13%
FBXW7	chr4:153249457	NM_033632.3: c.1321C>T, p.Arg441Trp	37%
FGF12	chr3:191888259	NM_004113.5: c.415A>G, p.Lys139Glu	3%
FGFR3	chr4:1804668	NM_001163213.1: c.958G>A, p.Asp320Asn	35%
FOS	chr14:75748111	NM_005252.3: c.1127C>T, p.Thr376Met	22%
GLIS2	chr16:4387132	NM_032575.2: c.1190delG, p.Gly397fs	42%
HDAC2	chr6:114264517	NM_001527.3: c.1375delA, p.Thr459fs	14%
HDAC4	chr2:240024583	NM_006037.3: c.2107G>A, p.Gly703Arg	49%
KDM2B	chr12:121987375	NM_032590.4: c.566G>A, p.Arg189His	33%
KMT2A	chr11:118376815	NM_005933.3: c.10199C>T, p.Pro3400Leu	39%
KMT2B	chr19:36214865	NM_014727.2: c.3296delG, p.Gly1099fs	19%
KMT5C	chr19:55855127	NM_032701.3: c.466G>A, p.Gly156Ser	37%
LYN	chr8:56866474	NM_002350.3: c.721G>T, p.Glu241*	36%
MAST2	chr1:46500323	NM_015112.2: c.3982C>T, p.Arg1328Cys	40%
MBD1	chr18:47806311	NM_002384.2: c.52C>T, p.Arg18Cys	35%
NEK11	chr3:130947408	NM_024800.4: c.1436A>G, p.Tyr479Cys	21%
NEK8	chr17:27068442	NM_178170.2: c.1903T>C, p.Tyr635His	20%
NOTCH1	chr9:139399976	NM_017617.4: c.4372G>A, p.Ala1458Thr	36%
NR3C2	chr4:149041372	NM_000901.4: c.2578G>A, p.Val860Ile	41%
PBRM1	chr3:52696241	NM_018313.4: c.436C>T, p.Arg146*	38%
PDS5B	chr13:33344579	NM_015032.3: c.3954delA, p.Lys1318fs	18%
PHF8	chrX:54011458	NM_015107.2: c.2332C>T, p.Arg778Trp	56%
PIK3R2	chr19:18273884	NM_005027.3: c.1217G>A, p.Arg406His	35%
PKHD1	chr6:51613046	NM_138694.3: c.9368C>T, p.Ala3123Val	44%
PKP2	chr12:32974415	NM_004572.3: c.2020G>A, p.Val674Met	36%
PRDM15	chr21:43277389	NM_022115.4: c.1279G>A, p.Glu427Lys	33%
PRKACB	chr1:84647916	NM_002731.3: c.142A>G, p.Lys48Glu	37%
PRKDC	chr8:48805713	NM_006904.6: c.3833G>A, p.Arg1278His	34%
PRSS1	chr7:142459627	NM_002769.4: c.203G>A, p.Arg68His	35%
PTGS1	chr9:125148757	NM_000962.3: c.1042G>A, p.Val348Met	33%
PTPRJ	chr11:48166643	NM_002843.3: c.2878T>C, p.Tyr960His	6%
RET	chr10:43612149	NM_020630.4: c.2254T>C, p.Tyr752His	10%
SAV1	chr14:51111571	NM_021818.3: c.697C>T, p.Arg233*	42%

OncoSeq was developed and its performance characteristics determined by the Michigan Center for Translational Pathology. It has not been cleared or approved by the FDA. The laboratory is regulated under CLIA as qualified to perform high-complexity testing. This test is used for clinical purposes.

©2021 Cobain EF. *JAMA Oncology*.

All patients participating in Mi-ONCOSEQ had summary of clinically actionable genomic alterations summarized in the report format above, highlighting somatic point mutations (including tumor mutational burden), somatic indels, copy- number alterations, gene fusions, genes with outlier expression, and pathogenic germline variants for disclosure. Cases presented at precision medicine tumor board (PMTB) met one of the following criteria: 1) highly clinically actionable alteration for which sequencing-directed therapy (SDT) could be considered, 2) change in cancer diagnosis, or 3) rare or unexpected NGS finding of significant clinical or scientific interest. The PMTB included pediatric and adult oncologists, geneticists, pathologists, biologists, bioinformaticians, bioethicists, genetic counselors, study coordinators, and ad hoc expertise.

©2021 Cobain EF. *JAMA Oncology*.

eFigure 2. Diverse Metastatic Cancer Types Represented in the MET1000 Cohort and Biopsy Sites

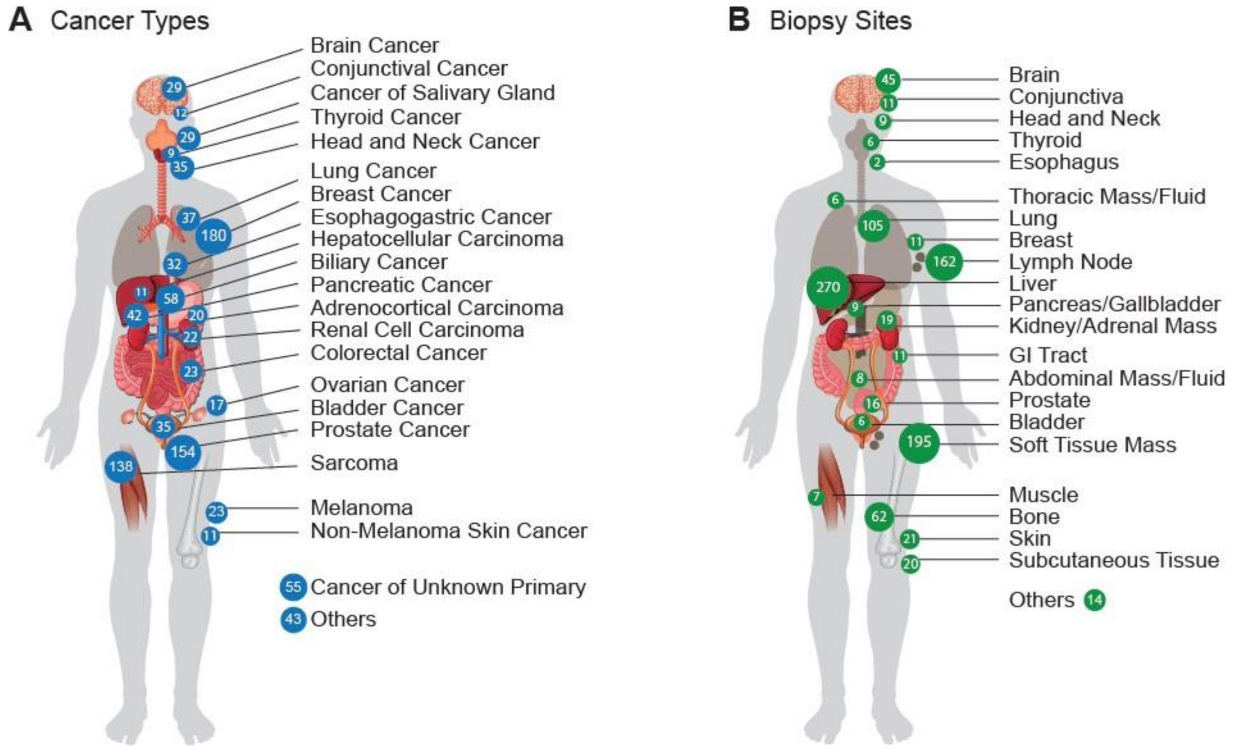

A. Number of each cancer type sequenced in the study.
B. Number of metastatic biopsies by site.

©2021 Cobain EF. *JAMA Oncology*.

eFigure 3. Time on Treatment for Patients With Exceptional Response to Sequencing-Directed Therapy

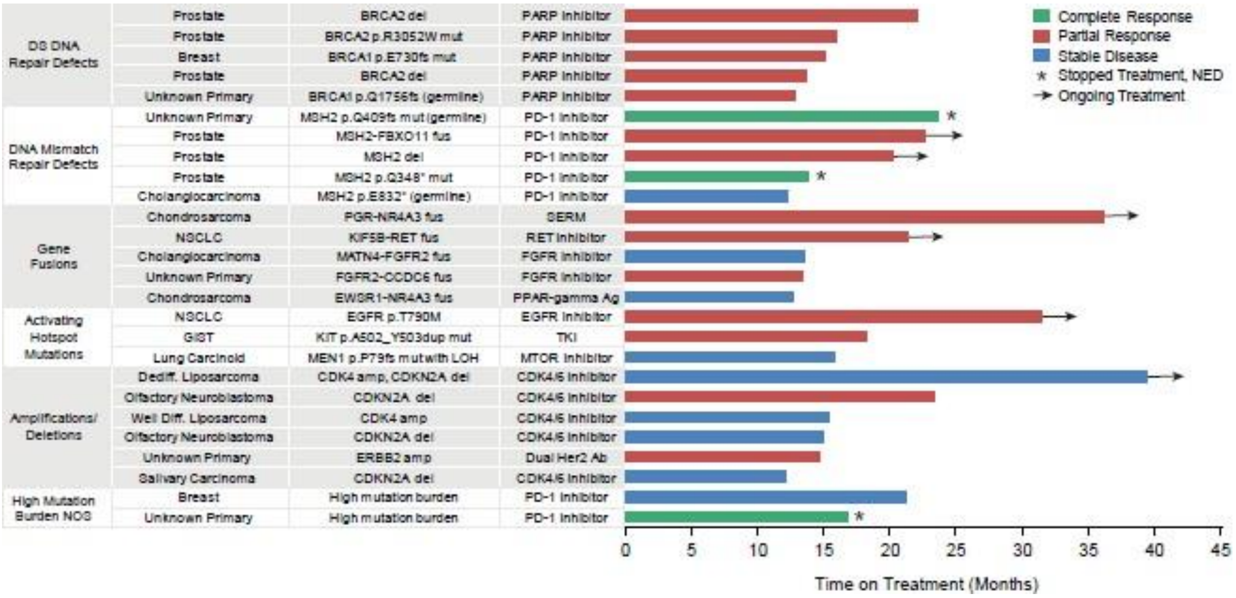

Patients are classified by disease type, clinically actionable genomic alteration, sequencing-directed therapy administered, and best response to therapy. Three patients had complete response which is ongoing despite cessation of therapy; NED, no evidence of disease. Six patients were receiving ongoing treatment at the time of data analysis. NSCLC, non-small cell lung cancer; GIST, gastrointestinal stromal tumor; mut, mutation; del, deletion; fs, frameshift; fus, fusion; LOH, loss of heterozygosity; amp, amplification; SERM, selective estrogen receptor modulator; TKI, tyrosine kinase inhibitor.

©2021 Cobain EF. *JAMA Oncology*.

eFigure 4. Baseline and Subsequent Computed Tomography Images From 4 Patients Who Had Exceptional Response to Sequencing-Directed Therapy

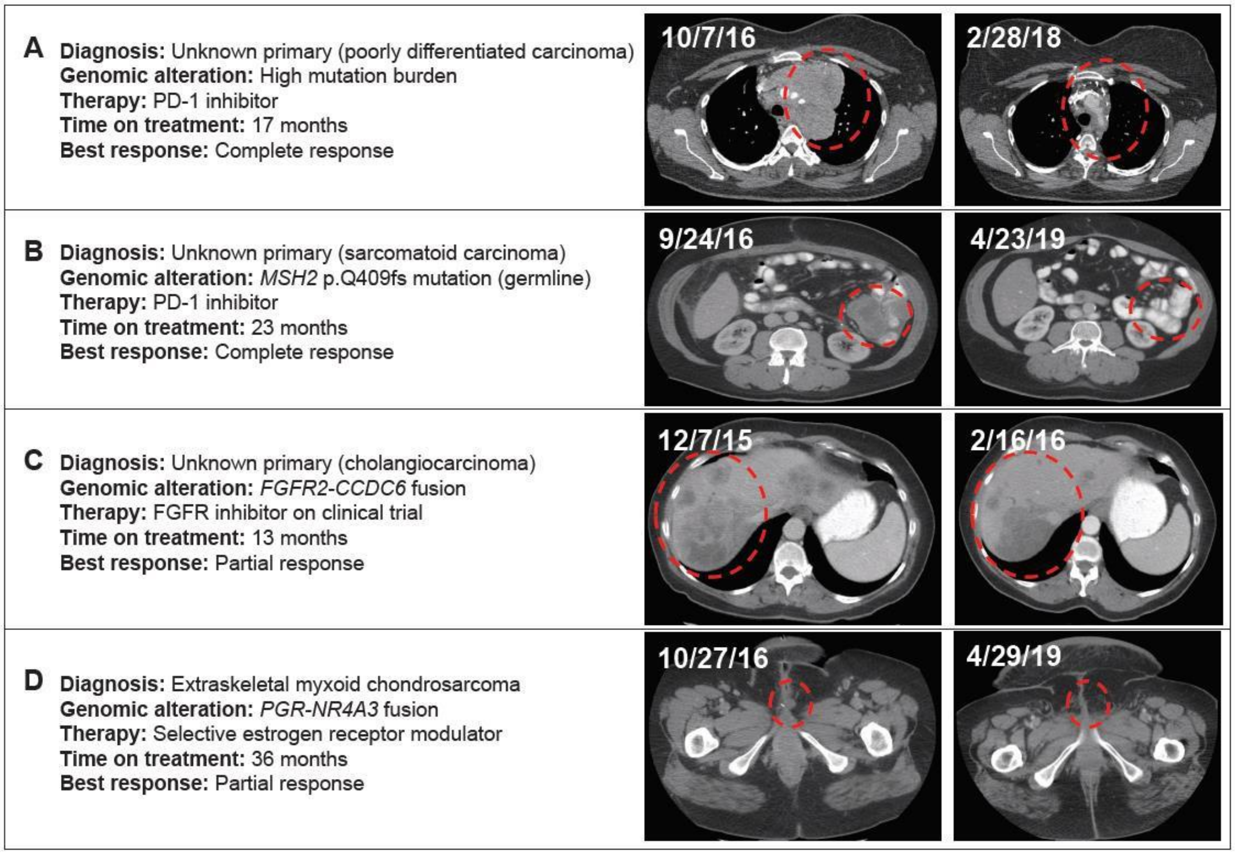

A. Patient with mediastinal mass of unknown primary origin (poorly differentiated carcinoma) diagnosed in July 2016 with high tumor mutation burden identified on genomic profiling who had exceptional response to single-agent PD-1 inhibitor therapy. The patient discontinued PD-1 inhibitor after 17 months due to complete response. **B.** Patient with sarcomatoid carcinoma of unknown origin (left upper quadrant abdominal mass) identified on genomic profiling to have a PGV in *MSH2* who had exceptional response to single-agent PD-1 inhibitor therapy. The patient discontinued PD-1 inhibitor therapy after 23 months due to complete response. **C.** Patient with unknown primary cancer involving liver, bone, and lung identified on genomic profiling to have cholangiocarcinoma with *FGFR2-CCDC6* fusion who had exceptional response to FGFR inhibitor administered on clinical trial lasting 13 months. **D.** Patient with extraskeletal myxoid chondrosarcoma involving bilateral inguinal regions with lung metastases, diagnosed during pregnancy. Genomic profiling identified a gene fusion involving the progesterone receptor (*PGR-NR4A3*) for which a selective estrogen receptor modulator was prescribed. Patient had partial response (near complete response) lasting 36 months which is ongoing.

©2021 Cobain EF. *JAMA Oncology*.

eFigure 5. Time on Treatment for Patients Receiving Therapy Based on a Pathogenic Germline Variant

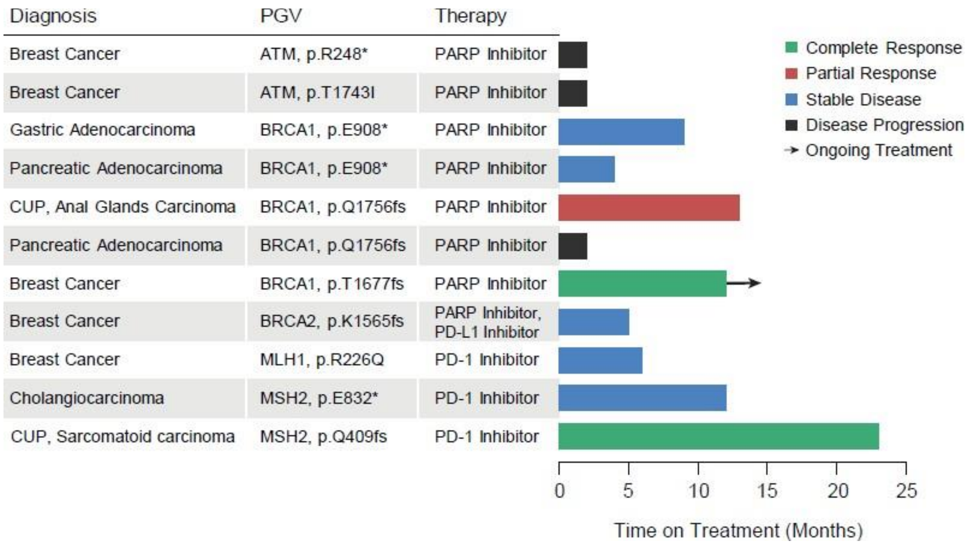

Eleven patients with clinical follow up received subsequent therapy on the basis of a pathogenic germline variant (PGV) identified, 10 of which were discovered by Mi-ONCOSEQ testing (one patient was previously known to have a PGV in *BRCA1*). Six patients achieved clinical benefit (time on treatment ≥ six months). One patient receiving a PARP inhibitor for a PGV in DNA repair and one patient receiving an immune checkpoint inhibitor for a PGV identified in mismatch repair achieved complete responses. Arrow indicates ongoing therapy. For two patients with cancer of unknown primary (CUP), one was reclassified following sequencing as an anal glands carcinoma and the other was best classified following sequencing as a sarcomatoid carcinoma. fs, frameshift; * indicates nonsense mutation (resulting in stop codon).

©2021 Cobain EF. *JAMA Oncology*.

Please see corresponding Excel spreadsheets for eTables 1-5

eTable 1. Gene List of the Mi-ONCOSEQ Targeted Panel

List of 1,700 genes included in the OncoSeq targeted panel with those genes reported as pathogenic germline variants (PGVs) designated when identified in both tumor and normal samples.

eTable 2. MET1000 Cohort Demographics and Sequencing Information

Case information including age, gender, biopsy site, type of tissue sequenced (frozen or formalin-fixed paraffin embedded (FFPE)), cancer diagnosis prior to Mi-ONCOSEQ enrollment, cancer diagnosis post- sequencing, primary tumor site, time elapsed between cancer diagnosis and enrollment in Mi-ONCOSEQ study, receipt of systemic therapy prior to Mi-ONCOSEQ enrollment, next-generation sequencing platform (whole-exome sequencing (WES) or targeted panel (OncoSeq)), tumor exome, normal exome, capture transcriptome and polyA libraries and designation of cases presented at precision medicine tumor board (PMTB). M, male; F, female; Y, yes; N, no; Mo, months.

eTable 3. Informative Molecular Events Identified in the MET1000 Cohort

Table indicates cancer type, gene of interest, chromosomal location, and type of informative molecular event. Corresponding loss of heterozygosity (LOH) events are also noted. The clinical tier for each molecular event is also indicated. Mut, Mutation; Del, homozygous deletion; Amp, amplification; Germ, germline; Fus, gene fusion; Exp, expression concordant with gene amplification; Dx, markers for cancer of unknown primary origin or change of diagnosis; Virus, viral pathogen.

eTable 4. Detailed Treatment Information for Patients in MET1000 Cohort Receiving Sequencing-Directed Therapy

eTable indicates date of enrollment, cancer type, actionable molecular alteration identified, and type of sequencing-directed therapy (SDT) administered, including whether or not therapy was administered on clinical trial (T), off-label (O), or on-label (L). Also noted is the duration of time patients were treated with SDT and best clinical response including progressive disease (PD), stable disease (SD), partial response (PR), complete response, or not applicable (N/A). Also included is the date of patient death or enrollment in hospice (if known) as well as duration of time between patient receipt of SDT and death or hospice enrollment.

eTable 5. Detailed Information Regarding Pathogenic Germline Variants Identified in the MET1000 Cohort

Table summarizes the cancer type in which PGV was identified, gene of interest, specific chromosomal location of PGV, corresponding protein change, and link to entry in ClinVar database. Also denoted is PGV clinical tiering information, with Tier 1 corresponding to PGVs associated with highly penetrant cancer predisposition syndromes, Tier 2 corresponding to PGVs associated with moderately penetrant cancer predisposition syndromes, Tier 3a corresponding to a carrier status which may be associated with increased risk of cancer or a lymphoproliferative disorder in a homozygous state, and Tier 3b corresponding to carrier status with no known cancer risk at present. Presence of a loss of heterozygosity (LOH) and whether or not the PGV was known prior to the patient's enrollment in the Mi-ONCOSEQ study is also indicated.

©2021 Cobain EF. *JAMA Oncology*.

eTable 6. Detailed Information Regarding Patients With CUP in the MET1000 Cohort

CASE	DIAGNOSIS AT ENROLLMENT	DIAGNOSIS AFTER SEQUENCING	# OF PRIOR LINES OF THERAPY	SITES OF DISEASE
MO_1075	CUP, Poorly differentiated carcinoma	CUP	1	mediastinal mass, bone
MO_1086	CUP	CUP	1	lung, bone, lymph node
MO_1092	CUP	CUP	3	lung, lymph node
MO_1123	CUP, Mucinous adenocarcinoma unknown primary	Pancreatic Cancer	0	pancreas, lung
MO_1141	CUP, SCC	CUP	1	lymph node, bone
MO_1175	CUP	Pancreatic Cancer	3	liver
MO_1197	CUP, SCC of unknown primary	CUP	2	lymph node
MO_1217	CUP, Undifferentiated Malignant Neoplasm	CUP	0	lymph node
MO_1223	CUP, Squamous cell carcinoma, NOS	CUP	0	lymph node
MO_1274	CUP	CUP	1	liver
MO_1284	CUP, Adenocarcinoma, Favor Lung primary	Lung Cancer	0	lung, liver, lymph node
MO_1285	CUP	Lung Cancer	0	lung, lymph node, bone
MO_1342	CUP	Esophageal Carcinoma	0	mediastinal mass, bone
MO_1363	CUP, Poorly differentiated carcinoma, NOS	CUP	1	lymph node
MO_1379	CUP	Ovarian Cancer	2	lung, lymph node, liver, pelvis
MO_1383	CUP	CUP	0	liver, lung
MO_1407	CUP	Renal Cell Carcinoma	2	lung, bone
MO_1419	CUP	CUP	2	lymph node, liver, mesentery
MO_1428	CUP	Cholangiocarcinoma	1	liver, lymph nodes
MO_1458	CUP	Serous Ovarian Cancer	0	periteonal carcinomatosis with malignant ascites
MO_1518	CUP	Cholangiocarcinoma	0	liver, lung, bone
MO_1542	CUP	Lung Adenocarcinoma	0	lymph node
MO_1563	CUP, SCC of unknown primary	CUP	0	pancreas, liver, spleen, omentum, peritoneum
MO_1597	CUP	Intrahepatic Cholangiocarcinoma	0	liver
MO_1611	CUP	CUP	0	lymph node, brain
MO_1634	CUP, SCC of unknown primary	Likely Head and Neck SCC	0	liver, bone
MO_1638	CUP	Lung Adenocarcinoma	1	lymph node
MO_1648	CUP	Breast Cancer	0	lymph node
MO_1677	CUP, Undifferentiated carcinoma of unknown primary	CUP	3	lung, peritoneum, pelvis
MO_1682	CUP	Cervical Squamous Cell Carcinoma	0	lymph node
MO_1692	CUP	Undifferentiated Gastric Carcinoma	0	malignant ascites
MO_1704	CUP	CUP	1	mediastinal mass, lymph node
MO_1783	CUP	Peritoneal Mesothelioma	0	liver, peritoneum
MO_1816	CUP	CUP	0	omentum, malignant ascites
MO_1847	CUP	Hepatocellular Carcinoma	0	liver, lymph node
MO_1897	CUP, Poorly differentiated malignant neoplasm	Amelanotic Melanoma	0	liver

©2021 Cobain EF. *JAMA Oncology*.

MO_2020	CUP, Poorly differentiated malignant neoplasm	CUP	0	liver, lymph node
MO_2026	CUP	Epithelioid Type Angiomyolipoma	0	kidney
MO_2050	CUP	Bladder Urothelial Carcinoma	0	pelvis, bone
MO_2078	CUP, Well differentiated neuroendocrine neoplasm	CUP	3	liver, bone
TP_2002	CUP	Breast Cancer	1	liver, omentum, malignant ascites, pleura
TP_2290	CUP	CUP	0	colon, lung, adrenal
TP_2311	CUP	CUP	0	adrenal
TP_2319	CUP	Colon Signet Ring Cell Adenocarcinoma	1	omentum, peritoneum
TP_2329	CUP, Mesenchymal undifferentiated round cell sarcoma	Solitary fibrous tumor (SFT)	0	kidney
TP_2423	CUP, SCC of unknown primary	CUP	0	diaphragmatic mass, pre-cardiac mass
TP_2450	CUP	CUP (No RNA lib)	0	liver
TP_2491	CUP	Male Breast Carcinoma (Triple Negative)	0	submental mass, lymph node, bone
TP_2500	CUP	CUP	0	lymph node, bone, lung
TP_2526	CUP	Endometrial Endometrioid Adenocarcinoma	4	lymph node, retroperitoneum, bone, liver, brain
MO_1507	CUP, small cell neuroendocrine	CUP	1	liver, lymph node, pleura
MO_1708	CUP, Adenocarcinoma	Perianal adenocarcinoma	0	lymph node
TP_2180	CUP	CUP	4	lymph node, skin
TP_2257	CUP, Sarcomatoid carcinoma	CUP	0	intra-abdominal mass
TP_2417	CUP, Neuroendocrine	CUP	2	liver

CHANGE OF DIAGNOSIS

CASE	DIAGNOSIS AT ENROLLMENT	DIAGNOSIS AFTER SEQUENCING
MO_1288	Breast cancer	Ovarian Cancer
MO_1774	Rhabdomyosarcoma	Renal Cell Carcinoma
MO_2057	Pancreatic Neuroendocrine Tumor	Cholangiocarcinoma
TP_2448	Synovial Cell Sarcoma	Solitary Fibrous Tumor

Table summarizes those patients who entered Mi-ONCOSEQ with diagnosis of cancer of unknown primary (CUP) origin and post-sequencing diagnosis. Also included are four patients that entered Mi-ONCOSEQ with a presumed known tissue of origin, where diagnosis was subsequently changed post-sequencing. For all patients with CUP, number of lines of systemic therapy prior to Mi-ONCOSEQ enrollment and sites of disease are indicated.

©2021 Cobain EF. *JAMA Oncology*.

eTable 7. Proportion of Patients Receiving Sequencing-Directed Therapy by Year of Enrollment

Year of Enrollment	Total Patients Enrolled	Number Initiating SDT (% of Total Patients Enrolled)
2012	79	7 (8.9%)
2013	137	5 (3.6%)
2014	144	15 (10.4%)
2015	140	10 (7.1%)
2016	199	46 (23.1%)
2017	265	42 (15.8%)
2018	38	6 (15.8%)

Increases in proportion of patients receiving sequencing-directed therapy (SDT) in years 2016-2018 corresponded with increased availability of clinical trials (ASCO TAPUR, NCI MATCH) enrolling on the basis of the presence of a genomic biomarker.

©2021 Cobain EF. *JAMA Oncology*.
